# Longitudinal survey of two serotine bat (*Eptesicus serotinus*) maternity colonies exposed to EBLV-1 (European Bat Lyssavirus type 1): Assessment of survival and serological status variations using capture-recapture models

**DOI:** 10.1371/journal.pntd.0006048

**Published:** 2017-11-17

**Authors:** Emmanuelle Robardet, Christophe Borel, Marie Moinet, Dorothée Jouan, Marine Wasniewski, Jacques Barrat, Franck Boué, Elodie Montchâtre-Leroy, Alexandre Servat, Olivier Gimenez, Florence Cliquet, Evelyne Picard-Meyer

**Affiliations:** 1 ANSES, Nancy Laboratory for Rabies and Wildlife–WHO Collaborating Centre for Research and Management in Zoonoses Control, OIE Reference Laboratory for Rabies, European Union Reference Laboratory for Rabies, European Union Reference Laboratory for Rabies Serology—Bâtiment H, Technopôle Agricole et Vétérinaire, CS, France; 2 CPEPESC Lorraine, Neuves-Maisons, France; 3 CEFE UMR 5175, CNRS, Université de Montpellier, Université Paul-Valéry Montpellier, EPHE, France; Wistar Institute, UNITED STATES

## Abstract

This study describes two longitudinal serological surveys of European Bat Lyssavirus type 1 (EBLV-1) antibodies in serotine bat (*Eptesicus serotinus*) maternity colonies located in the North-East of France. This species is currently considered as the main EBLV-1 reservoir. Multievent capture-recapture models were used to determine the factors influencing bat rabies transmission as this method accounts for imperfect detection and uncertainty in disease states. Considering the period of study, analyses revealed that survival and recapture probabilities were not affected by the serological status of individuals, confirming the capacity of bats to be exposed to lyssaviruses without dying. Five bats have been found with EBLV-1 RNA in the saliva at the start of the study, suggesting they were caught during virus excretion period. Among these bats, one was interestingly recaptured one year later and harbored a seropositive status. Along the survey, some others bats have been observed to both seroconvert (i.e. move from a negative to a positive serological status) and serorevert (i.e. move from a positive to a negative serological status). Peak of seroprevalence reached 34% and 70% in site A and B respectively. On one of the 2 sites, global decrease of seroprevalence was observed all along the study period nuanced by oscillation intervals of approximately 2–3 years supporting the oscillation infection dynamics hypothesized during a previous EBLV-1 study in a *Myotis myotis* colony. Seroprevalence were affected by significantly higher seroprevalence in summer than in spring. The maximum time observed between successive positive serological statuses of a bat demonstrated the potential persistence of neutralizing antibodies for at least 4 years. At last, EBLV-1 serological status transitions have been shown driven by age category with higher seroreversion frequencies in adults than in juvenile. Juveniles and female adults seemed indeed acting as distinct drivers of the rabies virus dynamics, hypothesis have been addressed but their exact role in the EBLV-1 transmission still need to be specified.

## Author summary

A multi-annual survey of two serotine bat (*Eptesicus serotinus*) maternity colonies previously found exposed to European Bat Lyssavirus type 1 (EBLV-1) was assessed using capture-recapture methodology. The two roosting site were located in the North-East of France. Animals were trapped, banded, and blood samples were collected to study their status regarding EBLV-1 exposure. Using capture-recapture models, the authors found that seropositive status of bats did not affect the survival abilities of individuals. Seroprevalence of EBLV-1 antibodies within the study showed an oscillation interval of approximately 2–3 years and a higher evidence of contact with the virus in summer than in spring. The maximum duration observed between successive positive serological statuses in the bat population also demonstrated a survival for at least 4 years after the exposition. This study confirms the ability of bats to survive despite circulation of lyssaviruses within the colony. Bats could indeed provide a valuable key to improving human health, currently facing numerous zoonotic epidemic issues.

## Introduction

Chiroptera is the second largest order of mammals after Rodentia. They have a worldwide geographical range, with the exception of Antarctica and are represented by more than 1 100 different species [1], of which 36 are found in Europe. Of these 36, 34 are reported in France, and all of them are strictly protected by national [2, 3] and international [4] legislation as they are sensitive to the destruction of their habitat. With their long lifespan regardless of their size, their unique flying ability as a mammal, and their overactive immune system, they are considered exceptional mammals and such fundamental innate abilities have recently attracted the interest of the scientific community [5–8]. More than 200 viruses have been associated with bats [9].They were recently discovered to be potentially at the origin of the Zaire Ebola virus [10, 11], and they have also been linked to other illnesses related to coronaviruses (Severe Acute Respiratory Syndrome, Middle Eastern Respiratory Syndrome), filoviruses (Ebola and Marburg), henipaviruses (Hendra and Nipah), and Lyssaviruses [12–14].

Rabies is a severe and lethal disease transmitted by the saliva of an infected animal through bite, dogs being the main source of human infection. The current lyssavirus taxonomy includes 14 lyssavirus species of the Rhabdoviridae family, order Mononegavirales [15], of which 12 species have been isolated in bats (reservoirs of the Mokola virus (MOKV) and Ikoma lyssavirus (IKOV) still remain to be identified [16, 17]). Phylogenetic analyses suggest that all these lyssaviruses have bat origins [18–20]. The number of recorded species will certainly increase in the future as suggested by the latest isolations of Gannoruwa Bat Lyssavirus in a fruit bat (*Pteropus medius*) in Sri Lanka, a new candidate in the formal classification of lyssaviruses [21].

In Europe, bat lyssavirus was documented for the first time in 1954 in Hamburg, Germany [22]. From 1977 to 2016, 1,175 bat lyssavirus cases were recorded from the North to the South of the continent [23]. To date, 4 different lyssavirus species have been isolated in European bats. Initially, European bat lyssaviruses were genetically described into 2 different groups named European bat lyssavirus type 1 (EBLV-1) and European bat lyssavirus type 2 (EBLV-2) [24]. Recently, 2 new lyssavirus species represented by the Bokeloh Bat Lyssavirus (BBLV) located in Germany and in France [25, 26] and the West Caucasian Bat Virus (WCBV) located in southern Russia [27] have been identified. A putative Lleida bat virus was detected in Spain in *Miniopterus schreibersii* but does not yet have a taxonomic status [28]. Most European bat cases have been recorded as belonging to EBLV-1 (>95%), which is associated with the serotine bat, *Eptesicus serotinus* [29], and with *E*. *isabellinus* in Spain, a sibling species of *E*. *serotinus* [30]. EBLV-1 molecular characterization has separated this species into 2 sublineages, EBLV-1a and EBLV-1b [31]. Lineage 1a shows a western-eastern European distribution from Russia to central France, while variant 1b exhibits a southern-northern European distribution from Spain to Denmark [32]. Except for 5 EBLV-2 cases in Pond bats (*Myotis dasycneme*) in the Netherlands [33], all other EBLV-2 cases were isolated from Daubenton’s bats (*Myotis daubentonii)* within a distribution area including the Netherlands, United Kingdom, Switzerland, Germany and Finland [34–36]. Among this viruses, only EBLV-1 and EBLV-2 have been associated with human cases with two identified case per virus species [37].

In France, bat lyssavirus was identified for the first time in 1989 in the Lorraine region (North-East France) (Briey and Bainville) and a bat rabies surveillance program was consequently initiated [38]. Epidemiosurveillance and research programs to estimate the public health risks associated with the infection of native bats by Lyssavirus were then strengthened following the report of the French Ministry of Agriculture [39], leading to the consolidation of the network involving both local veterinary services and the French National Bat Conservation Network (SFEPM). From 1989 to present, 78 bat lyssavirus cases—75 EBLV-1 cases in common serotine bats, 1 EBLV-1 case in common pipistrelle *(Pipistrellus pipistrellus)* and 2 cases of BBLV in Natterer's bats *(Myotis nattereri)—*have been diagnosed in France (E. Picard-Meyer, under revision) and the issue of seasonality in the probability of detecting cases has been raised recently [40]. To gain a better understanding of virus transmission, active surveillance programs during population monitoring were set up in addition to the passive surveillance program. As shown in the synthesis made by Picard-Meyer for the 2004–2009 period, the sampling of such programs involved blood and saliva samples from more than 300 bats on 18 sites [41]. In such cross-sectional surveys, the bats were sampled on various sites throughout France and no data from marked individuals were available to allow a longitudinal study. This study proposes the first longitudinal survey of EBLV-1 in mono-specific serotine colonies, the main bat species found infected by this lyssavirus. Multi-state models, a category of capture-recapture analysis, have been used to attempt explaining EBLV-1 virus exposure. Originally developed for estimating the abundance of animal populations, capture-recapture methods have recently attracted attention in the field of veterinary epidemiology [42, 43]. When appropriate data are available, capture-recapture models can also be used directly to estimate disease-associated mortality and epidemiological parameters, such as infection and recovery rates [44]. In this study, as the serological status of individuals could change, data were analyzed using a multistate capture-recapture approach [45]. Multistate models indeed allow individuals in a population to be distributed across multiple sites or among different disease states [46, 47]. More precisely, we used multievent capture-recapture models [48], an extension of multi-state models, to determine the factors influencing bat rabies transmission while accounting for imperfect detection and uncertainty in disease states. Survival, capture, transition and judgment probabilities were assessed by hypothesizing, based on lyssaviruses literature, that EBLV-1 exposure in bat maternity colony was driven differently according to the age of individuals and the period of time. To our knowledge, this is the first attempt of describing EBLV-1 circulation in its reservoir, over time, and by such approach and methodology.

## Materials and methods

### Study sites

**Fig 1:**
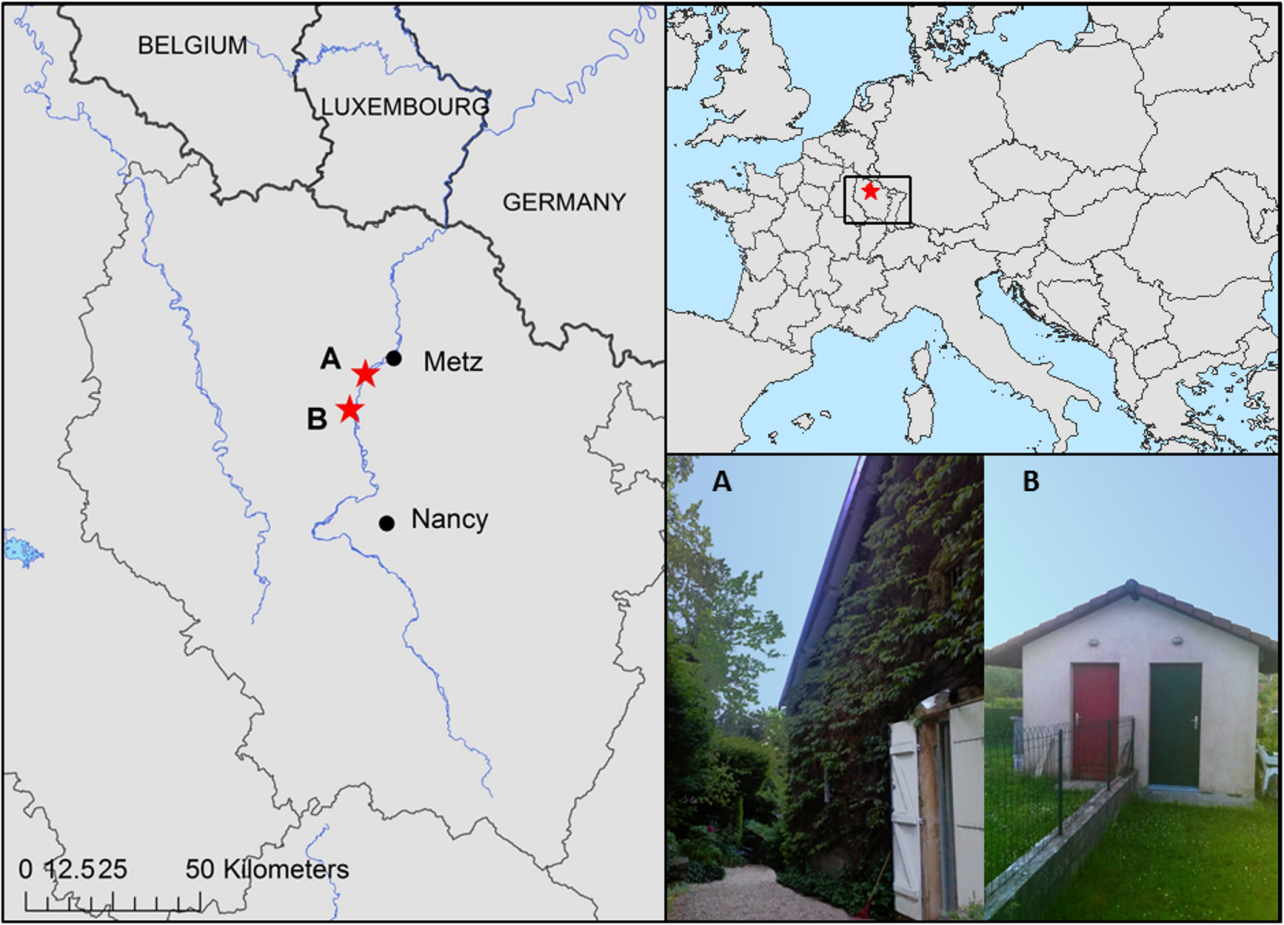
Geographic location of roost sites A (Ancy-sur-Moselle) and B (Pagny-sur-Moselle).

Two maternity roost sites of serotine bat colonies located in the East of France (Fig 1) were monitored. The sites were located in Universal Transverse Mercator (UTM) 32U zone in villages bordered by Moselle River and surrounded by hardwood forest, cropland and grassland. The climate is semi-continental and the landscape relatively flat with altitude ranging from 167 to 374 meters. Site A was the roof of a house in Ancy-sur-Moselle in the Moselle department, while site B—8.5 km from site A—was the garden shed of a house in Pagny-sur-Moselle in the Meurthe-et-Moselle department. Both sites were chosen following the detection of bat cadavers in 2009 and 2012 for site A and 2011 for site B (E. Picard-Meyer, under revision). On site A, 6 dead animals in 2009 and one dead animal in 2012 tested positive for lyssavirus with reference techniques [49–51]. On site B, 2 dead individuals tested positive in 2011. The infection was shown to be caused by the EBLV-1b variant, which is endemic in the region. They were the first detection of EBLV-1 infections in these municipalities.

### Field data collection

Capture-recapture sessions were completed in summer (July and August) and spring (May) between 2009 and 2015 (during 7 years) for site A, and between 2011 and 2015 (during 5 years) for site B. Capture sessions were organized by the French Agency for Food, Environmental and Occupational Health & Safety (ANSES) and the Commission for the Protection of Water, Heritage, Environment, Subsoil and Chiroptera (CPEPESC) of Lorraine Region, the naturalist association in charge of the study and protection of bats in the region. Residents provided full informed consent to have their residences used in the study. The trapping session dates were set up to avoid disturbing the bats during the parturition period.

Captures were held at nightfall, when serotine bats are known to leave their roost to forage. Harp traps were used because they are the most suitable device when a large number of animals can be expected [52]. Moreover, these traps are considered the most effective for capturing bats without harming them [53]. Traps were placed at the exits of the roof from where the bats usually emerged. To avoid injury, they were handled carefully and firmly by trained people wearing gloves and adequately vaccinated against rabies. As soon as the bats were removed from the traps, they were placed temporarily in cotton bags and then held in the palm of the hand with fingers curled around the body [54]. The sex and age class were recorded for each animal and biological samples collected.

Dry synthetic fiber swabs (classiqSwabs, COPAN, France) were soaked with saliva to assess EBLV-1 virus excretion as well as viral RNA detection. Blood was collected from the antebrachial vein along the propatagium, and more recently on the uropatagium, a method found more effective, to evaluate the serological status of each individual with respect to EBLV-1 exposure. Blood was collected using filter paper as described by Wasniewski et al. [55] and subsequently stored at -16°C till the analysis. Swabs were stored in 0.3 mL of DMEM culture medium (Dulbecco’s minimum essential medium, Invitrogen, France) at -80°C for further testing in the laboratory. Lipped bat bands (split metal bat rings, PORZANAZTD, East Sussex, United Kingdom) positioned on the forearm were used to mark the animals [56]. Each bat captured was assigned a single record number, allowing for follow-up over the successive capture sessions. After sampling, all the bats were immediately released at the site of night-time capture. None of the bats appeared sick or were euthanized during the study period.

### Ethics statement

All the animals were handled in strict accordance with good animal practices and according to the EUROBAT guideline [57]. Field work and animal sampling were performed in accordance with French legislation. Because bats are protected species in France, prior formal authorization by the French Ministry of the Environment was granted for their trapping, handling, and sampling [58] and colony monitoring was undertaken following local authorization by the Prefect of the Lorraine Region [59]. In France and within the European Union, the legal framework for using under experimentation purposes is governed by Regulation 2010/63/EU of the European parliament and of the council of 22 September 2010 (applicable and translated in French in 2013) and handling of wildlife animal in the field does not require any prior specific ethical approval.

### Laboratory testing

#### Saliva analysis

One saliva sample was collected per trapped individual in order to assess if animals were excreting EBLV-1. Oral swabs (n = 320 for site A and 469 for site B) were analyzed by RTCIT (Rabies Tissue Culture Infection Test) on murine neuroblastoma cells (ATCC-CCL31) to detect any infectious lyssavirus [49, 60] and by RT-PCR to identify viral EBLV-1 RNA. Viral RNA was extracted from 200 μl of saliva sample using the Iprep Pure LineR Virus kit (Invitrogen, France) according to the manufacturer’s instructions. The conventional hnRT-PCR technique previously described by Picard-Meyer et al. [61] was used between 2009 and 2011 for site A and in 2011 for site B. This technique included universal primers (JW12-JW6) in the first round and specific primers of the lyssavirus species EBLV-1 (JW12-JEBL1) in the second round [61]. The conventional RT-PCR with specific rabies primers enabled an end-point of 10^−5.93^ Mouse Intracranial Median Lethal Doses (MICLD)50, while the end-point detection for MIT and RTCIT were, respectively, 10^−0.42^, and 10^−0.2^ MICLD50 [61]. One step Real-time SYBR Green RT-PCR described by Picard-Meyer et al. [62] was used for both sites between 2012 and 2015 using Pan-Lyssavirus primers (JW12-N165-146). This technique allows the detection of the nucleoprotein gene of all known lyssaviruses and has been shown rabies specific [63] and sensitive with a limit of detection (LOD95%) of 20 copies/μL of RNA [62]. Usual precautions for both conventional and real-time PCR were strictly followed in the laboratory to avoid false-positive results. Negative and positive controls were also used in each run to assess the reliability of PCR.

#### Blood sample analysis

Blood samples were collected to evaluate if animals had been exposed to EBLV-1. A modified Fluorescent Antibody Virus Neutralisation test (mFAVNt) [64] was performed with an EBLV-1b virus strain (ANSES, N°121411, France, 2000) in order to detect EBLV-1b-specific neutralizing antibodies in blood samples (n = 309 blood samples for site A and 389 for site B) [41]. Briefly, samples were tested in a threefold dilution on Baby Hamster Kidney (BHK)-21 cells (ATCC-CCL10) with a starting dilution of 1/27. Controls included uninfected BHK-21 cells, OIE positive dog serum [65], negative dog serum (ANSES collection) and back-titration of the specific EBLV-1 virus. Levels of virus-neutralizing antibodies were expressed in log D50. The threshold of antibody detection was calculated using the Spearman-Karber formula and set at 1.67 log D50 [41]. As the purpose of the study was to reliably identify positive individuals, the cut-off value of 1.67 (in logD50) has been chosen to reach specificity equal to 100%. Indeed the positive samples (having a titre equal to or above this cut-off value) detected by the modified FAVNt are 100% concordant with results obtained with the FAVNt [66].

### Statistical analysis

During each capture session, the captured animals were recorded and classified into different serological states concerning EBLV-1 neutralizing antibodies, “**S”** (“NEG”/ “POS”/ “INC”). The “**NEG**” state included EBLV-1 seronegative individuals, i.e. bats that had never been in contact with the virus, and consequently susceptible to future infection or previously exposed but with a non-detectable level of EBLV-1 antibodies. The “**POS**” state included EBLV-1 seropositive animals. Seropositive animals were defined as animals that had been in contact with the virus and seroconverted. This state included both bats that were potentially protected against infection by antibodies, and sick bats. Because the serological test was occasionally inconclusive (analysis not feasible), or no blood was sampled, an “**INC**” state was included. To address this particular issue and to allow the use of such a dataset, an extension of the multistate capture-recapture framework was used. This extension is known as the multievent model [47, 67]. When an individual is observed in the field, its status can still remain unknown or uncertain, e.g. sex status [68], reproductive status [69] but also epidemiological status [42, 47] and such a model accounts for uncertainties in the assessment of a state.

The different probabilities assessed during the study were as follows:

- Survival probability **(ϕ)**: the probability that an individual (with “POS” or “NEG” status) remains alive over a given period of time.
- Recapture probability **(p)**: the probability that a living individual (with “POS” or “NEG” status) is encountered during a capture session.
- Transition probability **(ψ)**: the probability that, over a given period of time, an individual “moves” from one serological state to another, i.e. from seronegative to seropositive status (“NEG” to “POS”) or from seropositive to seronegative status (“POS” to “NEG”), or remains in its current state.
- Judgment probability **(δ)**: the probability that an “INC” status individual had a seropositive (“POS”) or seronegative (“NEG”) status.

The models were fitted using the E-SURGE program [70]. Both sites were maternity colonies mainly composed of females with roost site fidelity and juvenile males leaving the colony at the end of their first summer [71], males were consequently discarded from the dataset to avoid bias in the survival analysis. Multiple capture sessions were conducted occasionally within the same season (between 2 and 5), and the detection/non-detection data were merged into a single capture session per year and season. Survival, recapture, transition and judgment probabilities were all computed by considering the serological status“S” (POS/NEG/INC) as explanatory variable. Age class **“a”** (juvenile/adult) was also considered as a potential explanatory variable for survival probabilities as juveniles could harbor higher mortality rate as demonstrated in serotine bat biology study [72]. Regarding serological transition probabilities, a previous EBLV-1 study suggested seasonal fluctuation in *Myotis myotis* colonies [73], we consequently hypothesized that serotine colony could by driveen by a comparable dynamic and included the season **“s”** (spring/summer) as explanatory variable. This study being the only known EBVL-1 longitudinal studies on serotine monospecific colonies, we also assumed based on classical bat rabies virus (RABV) studies that transmission rate could vary according the age [74] and included age class **“a”** (juvenile/adult) in candidates models. The year **“y”** and/or season **“s”** (spring/summer) effects and their interaction were considered with regard to recapture probabilities as weather variations are suspected to impact trapping efficiency. Possible interactions with the serological status were also assessed to determine whether there were any specific infection patterns. All model combinations to estimate survival, transition, capture and judgment probabilities fit accordingly.

Akaike's Information Criterion with a correction for small sample sizes (AICc) was used to assess the relative model fit. The model with the lowest AICc was selected as the model that fitted the data best [75]. When the ΔAICc was lower than 2 (Δi = difference between AICc and the lowest AICc value), the most parsimonious model was selected (i.e. the one with the fewest variables).

To compute antibody prevalence and its standard error, we used the traditional abundance estimate and corrected the number of animals that tested positive or negative in each session by the corresponding recapture probability [46]. To account for “INC” observations, bats were assigned a “POS” or “NEG” status using the Viterbi algorithm [76]. For each site, a logistic regression was used to assess the effect of season and year on the estimated prevalence. The number of positive and negatives cases was used as the response variable, and the AICc was used to compare models either incorporating or excluding time variables.

## Results

### Sampling, serological history and transitions

On site A, 15 capture sessions were undertaken between 2009 and 2015, corresponding to a total of 320 bat captures (including single captures and recaptures). The distribution of the number of captures and recaptures per year and season is presented in Table 1. Among the 214 marked animals, 81 individuals (38%) were recaptured once, 19 individuals (9%) were recaptured twice, 5 individuals (2%) were recaptured 3 times and 1 individual was captured 5 times within the study period. Within the studied 201 individuals were females (94%) and 13 were males (6%). All males but 2 were identified as juveniles. Both adults had a single capture history.

**Table 1:** Raw data summary: Total number of captures and recaptures in site A.

Site A	Number of 1st captures (marked animals)				Number of recaptures			
Time period	Total	juvenile males	juvenile females	adult females	Total	juvenile males	juvenile females	adult females
Summer 09	79	6	6	67	27	3	0	24
Spring 10	10	0	0	10	2	0	0	2
Summer 10	32	3	11	18	15	0	0	15
Spring 11	18	0	0	18	14	0	0	14
Summer 11	6	0	2	4	3	0	0	3
Spring 12	19	0	0	19	10	0	0	10
Summer 12	18	1	1	16	15	0	0	15
Spring 13	6	0	0	6	3	0	0	3
Summer 13	14	2	9	3	5	0	0	5
Spring 15	12	1	0	11	12	0	0	12
Total	214	13	29	172	106	3	0	103

By comparison, on site B, where 12 capture sessions were undertaken between 2009 and 2015, there was a total of 473 bat captures, single captures and recaptures combined. The distribution of the number of captures and recaptures per year and season is also presented in Tables 1 and 2. Among the 221 marked animals, 125 individuals (57%) were recaptured once, 60 individuals (27%) were recaptured twice, 36 individuals (16%) were recaptured 3 times, 21 individuals (10%) were recaptured 4 times, 7 individuals (3%) were recaptured 5 times and 1 individual were captured 7 times, 8 times and 9 times within the study period. 156 captured bats were females (71%) while 65 were males (30%). It should be noted that no other bat species have been identified within the study period, excepted one time where a *Miniopterus schreibersii* individual was trapped.

**Table 2:** Raw data summary: Total number of captures and recaptures in site B.

Site B	Number of 1st captures (marked animals)				Number of recaptures			
Time period	Total	juvenile males	juvenile females	adult females	Total	juvenile males	juvenile females	adult females
Summer 11	44	7	12	25	14	1	6	7
Spring 12	22	0	0	22	7	0	0	7
Summer 12	65	22	16	27	54	2	3	49
Spring 13	4	0	0	4	6	0	0	6
Summer 13	26	9	14	3	20	0	0	20
Spring 14	10	0	0	10	37	0	0	37
Summer 14	46	27	14	5	67	10	7	50
Spring 15	4	0	0	4	47	5	0	42
Total	221	65	56	100	252	18	16	218

Many different serological status histories were observed during the study (S1 Table). Thus, some animals evolved from a negative to positive status, some did the reverse, and occasionally some changed several times (See Supplementary material describing the frequencies of the different capture histories, inconclusive results were ignored for a better clarity). This process revealed that it was more frequent for a seropositive status to become seronegative than the opposite (Table 3). Indeed, 5 and 9 animals seroconverted (from NEG to POS) while 10 and 21 animals seroreverted (from POS to NEG) on sites A and B respectively.

**Table 3:** Serological transitions observed in multiple capture/recapture sessions of bats (inconclusive statuses are excluded and consecutive identical statuses were merged to improve clarity).

n individuals on site A	n individuals on site B	Serological transitions observed			
123*	108	NEG			
50**	56	POS			
5	9	NEG	POS		
10	21	POS	NEG		
1	6	NEG	POS	NEG	
1	1	POS	NEG	POS	
0	1	NEG	POS	NEG	POS
0	1	POS	NEG	POS	NEG

* one individual detected with EBLV-1rabies RNA in the saliva (potentially excreting the virus)

** one individual detected with EBLV-1RNA in the saliva; and one individual excreting the virus (both PCR and RTCIT were found positive).

When analyzing oral swabs, all the tested animals were found negative for RNA detection in the saliva, apart from 5 individuals all captured in July 2009 on site A. Viral RNA was detected during a first capture for 4 animals and during a second capture for one animal. Among the animals captured for the first time, 3 animals (2 females—one adult, and one juvenile—and one juvenile male) were only captured once. Among the two recaptured bats, one adult female was sampled again during the next capture session but was surprisingly found to be seronegative, with no RNA detection. The history of the second recaptured bat, found RNA-positive in the second capture of the same year, was notable. The adult female bat was indeed negative for both RNA and serology in July 2009 then, 3 days later, positive for RNA but inconclusive as to its serological status (the blood sample could not be assessed) and negative for virus excretion yet seropositive for EBLV-1 antibody detection in August 2010, meaning one year later saliva has been detected RNA-positive. All the virus isolation tests failed to detect a live virus except for one seropositive juvenile female captured only once in July 2009 on site A, just after the positive testing of dead bats. All the samples from site B were negative for the presence of an infectious virus. Individual movements between the 2 colonies, 8 kilometers away, were possible but difficult to quantify as only one female marked on site B in July 2012 was found on site A in August 2013.

### Survival, transition, capture and judgement probabilities

The best models for both sites A and B included effects for the year and season on recapture probabilities (Tables 4 and 5). No specific pattern was detected for survival probability on either site, neither age category nor serological status affecting survival. We found an effect of the interaction of serological status and age on the transition probability for site B, while the judgment probability depended on the serological status for site A only.

**Table 4:** Top ten ranked models along with a null model describing factors affecting survival (ϕ), recapture (p), transition (ψ), and judgment (δ) probabilities on site A. The best-ranked model is in bold.

Model	n Parameter	Deviance	AICc	ΔAICc
**ϕ(i), ψ(S), p(s.y), δ(S)**	**15**	**960.26**	**992.25**	**0**
ϕ(a), ψ(S), p(s.y), δ(S)	16	958.47	992.74	0.49
ϕ(S), ψ(S), p(s.y), δ(S)	16	960.10	994.37	2.12
ϕ(i), ψ(S), p(s.y), δ(i)	14	964.64	994.38	2.13
ϕ(a), ψ(S), p(s.y), δ(i)	15	962.86	994.85	2.60
ϕ(i), ψ(a.S), p(s.y), δ(S)	17	960.02	996.58	4.33
ϕ(i), ψ(s.S), p(s.y), δ(S)	17	960.03	996.59	4.34
ϕ(S), ψ(S), p(s.y), δ(i)	15	964.64	996.64	4.38
ϕ(a), ψ(a.S), p(s.y), δ(S)	18	958.23	997.11	4.86
ϕ(a), ψ(s.S), p(s.y), δ(S)	18	958.24	997.11	4.80
null model: ϕ(i), ψ(i), p(i), δ(i)	5	1022.65	1032.88	40.63

Note: AICc: Akaike’s information criterion corrected for a small sample size; ΔAICc: differences in AICc.

I = intercept; S: health status (POS, NEG, INC); s: season (spring/summer); y: year; a: age (juvenile/adult).

**Table 5:** Top ten ranked models along with a null model describing factors affecting survival (ϕ), recapture (p), transition (φ), and judgment (δ) probabilities on site B. The best-ranked model is in bold.

Model	n Parameter	Deviance	AICc	ΔAICc
**ϕ(i), ψ(a.S), p(s.y), δ(i)**	**14**	**1237.23**	**1265.23**	**0**
ϕ(S), ψ(a.S), p(s.y), δ(i)	15	1235.58	1265.58	0.55
ϕ(a), ψ(a.S), p(s.y), δ(i)	15	1236.83	1266.83	1.80
ϕ(i), ψ(a.S), p(s.y), δ(S)	15	1237.12	1267.12	2.08
ϕ(S), ψ(a.S), p(s.y), δ(S)	16	1235.53	1267.53	2.71
ϕ(i), ψ(s.S), p(s.y), δ(i)	14	1239.81	1267.81	2.58
ϕ(a.S), ψ(a.S), p(s.y), δ(i)	17	1234.47	1268.47	3.87
ϕ(a), ψ(s.S), p(s.y), δ(S)	16	1236.72	1268.72	3.89
ϕ(a.S), ψ(a.S), p(s.y), δ(S)	18	1234.43	1270.43	6.07
ϕ(S), ψ(S), p(s.y), δ(i)	13	1256.15	1282.15	16.74
null model: ϕ(i), ψ(i), p(i), δ(i)	5	1356.19	1366.38	99.81

Note: AICc: Akaike’s information criterion corrected for a small sample size; AICc: differences in AICc.

I = intercept; S: health status (POS, NEG, INC); s: season (spring/summer); y: year; a: age (juvenile/adult).

On site A, the best-ranked model indicated that the survival probability of female bats was 0.86 [0.76–0.93]. The transition probability from seropositive to seronegative was 0.99 [0.02–0.99] (a boundary estimate that was difficult to interpret) and 0.21 [0.09–0.40] from seronegative to seropositive. The probability of judging a positive result as positive was 1 while the probability of judging a negative result as negative was 0.81 [0.75–0.86].

On site B, the best-ranked model indicated that the survival probability of female bats was 0.78 [0.73–0.83]. The transition probability from seropositive to seronegative was 0.89 [0.53–0.98] and 0.25 [0.06–0.61] from seronegative to seropositive for adult female bats and 0.15 [0.07–0.27] and 0.05 [0.02–0.12] for juveniles female bats respectively.

### Evolution of corrected seroprevalence

**Fig 2:**
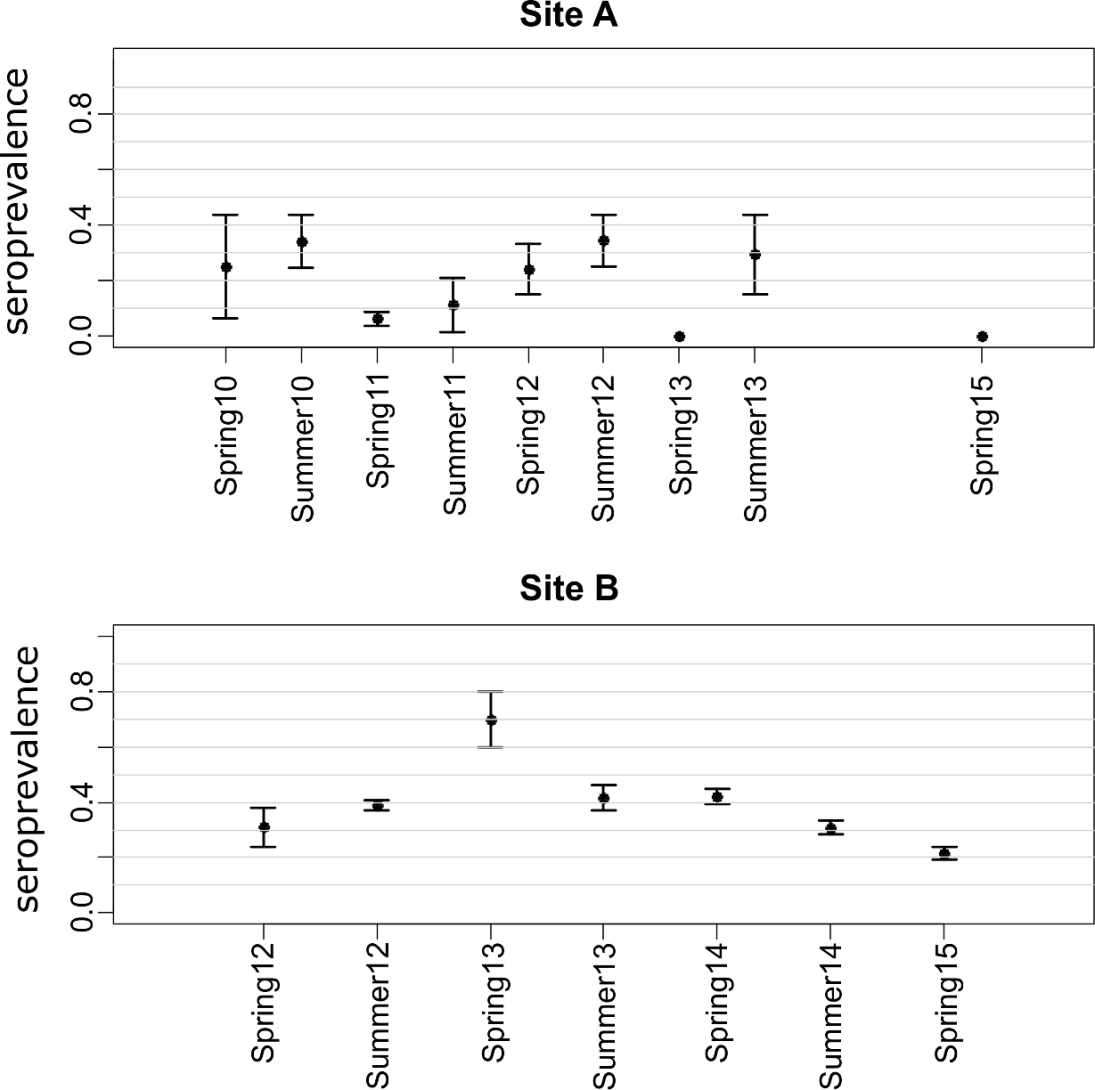
Evolution of corrected seroprevalence on sites A and B.

The evolution of corrected bat EBLV-1 seroprevalence on both sites A and B are presented in Fig 2. On site A, seroprevalence varied from 34% in summer 2010 [28.0–41.0] and summer 2012 [27.5–41.9] to 0% in spring 2013 [0–1.5] and spring 2015 [0–6.1]. The trend appeared to indicate a constant decrease in seroprevalence over time during the study, with an approximate 2–3 year oscillation interval (2011–2013). The logistic regression detected a significantly lower frequency of seropositive cases from 2011 to 2013 than in 2010 (OR_2011_ = 0.26 [0.19–0.37]; OR_2012_ = 0.65 [0.47–0.90]; OR_2013_ = 0.40 [0.29–0.55]) and a lower frequency of seropositive cases in spring than in summer (OR_spring_ = 0.46 [0.35–0.60]).

On site B, seroprevalence peaked at 70% [59.9–78.9] in spring 2013 then progressively decreased to reach 21.6% [11.5–34.9] in May 2015. The logistic regression did not detect any significant differences between seasons, but the frequency of seropositive cases in 2013 was higher than in 2012 (OR_2013_ = 2.40 [1.61–3.60]).

## Discussion

Most European bat rabies cases have been detected in serotine bats, currently considered to be the main EBLV-1 reservoir [40]. Despite its evident role in the transmission of EBLV-1, the modality and dynamics of transmission in serotine bats are still poorly understood. This work focuses on a unique long term capture-recapture study of two serotine bat maternity colonies exposed to EBLV-1. Bats infected with EBLV-1 were indeed detected in 2009 and 2012 on site A and in 2011 on site B. It should be noted that as marking a bat is strictly forbidden in Europe and in France requires a special authorization delivered by the Ministry of the Environment, such field studies are difficult to implement and limited in number, making them a source of precious data.

### Survival and recapture probabilities for seropositive and seronegative animals

The analysis of the two roost site colonies using multievent models within the study period did not evidence any impact of the serological status on individuals’ survival or recapture probabilities. This supports previous observations that bats could harbor exposure events without any impact on their mortality rate. These results are indeed comparable to the survival analysis computed for big brown bats (*Eptesicus fuscus*) affected by RABV in the United States [77] and for a *Myotis myotis* colony affected by EBLV-1 in Spain [73].

The detection rates of bats were also demonstrated to be uncorrelated to the serological status, indicating that seropositivity does not induce a potential behavioral change in bats that could impact the recapture probability. This finding supports the hypothesis that, in our study, observed seroprevalence of a capture session can be regarded as an unbiased estimation of the percentage of animals wo have been exposed to EBLV-1 in the colony. Recapture probabilities on both sites were shown to be affected by seasonal and annual variations. This temporal dependency could be due to changes in climate and weather conditions over seasons and years, known to affect the emergence of bats and consequently the effectiveness of captures using a harp trap [78].

### Serological transition scenarios and virus excretion

EBLV-1 virus-neutralizing antibodies have been found in various bat field studies, principally through single captures [41, 79, 80] or in successive captures of mono-specific colonies like *Myotis myotis* [73], *Eptesicus isabellinus* [81] and also in longitudinal studies of multi-species colonies [82–84]. In these previous studies, seroprevalence varied greatly according to the site location, species and time (month and year). This study, to our knowledge, is the first extensive longitudinal analysis of 2 mono-species serotine colonies, a species currently considered as the main EBLV-1 reservoir.

Our study demonstrated that individual serological transition scenarios are highly variable. We found seroconversions (from seronegative to seropositive), seroreversions (from seropositive to seronegative) in addition to occasional multiple seroreversions and seroconversions in succession (about 10/393 individuals). It should be noted that such multiple reversions could be questionable as they may also reflect limitations of the serological tests [85] performed furthermore on small amounts of blood. Globally, on both sites, seroreversions were more frequent than seroconversions, suggesting that the 2 studies could have occurred at the end of the rabies epizootic wave. This hypothesis could be supported by the fact that prior to the first established EBLV-1 case in July 2009, approximately 30 to 40 individuals were found dead by the house owner, but the animals were unfortunately not collected and analyzed. Previous longitudinal studies in Spain have shown the seropositive status of *Myotis Myotis* over 3 years [84]. The maximum length of time observed between positive serological statuses in our own study was 4 years (2 individuals on site A), suggesting the possible persistence of seropositivity over several years. In this study, cut-off level used to discriminate positive from negative animals was determined to minimize the risk of false positive results. This caution was taken to provide reliable identification of positive individuals and to avoid false conclusion in the statistical analysis, but could have resulted in an underestimation of seroprevalence and in low statistical power.

Lyssaviruses are excreted only at certain periods, and the chance of finding the virus or RNA in bat saliva during active surveillance field studies is relatively poor [41, 79]. Interestingly, and for the first time in EBLV-1 longitudinal study, 5 individuals from site A were found with viral RNA in saliva in July 2009 (four were sampled in the same capture session, in the beginning of July and 1 was sampled three days later), during the same period in which bat mortality was observed. Of the 5 positive samples, one was demonstrated as effectively infectious, showing that RNA in the mouth cavity can be concomitant to virus excretion. One initially seronegative animal was captured several times in succession. Saliva was found RNA positive 3 days later and, when captured again 1 year later, the animal was again found seropositive, supporting the hypothesis that seropositivity persists for a long time after infection or that alternate subclinical infection occurred. The risk of cross-contamination regarding the four RNA positive samples collected during the same capture session can’t be completely ruled out with certainty although usual precautions to avoid false-positive PCR results were strictly followed during bat handling and in the laboratory. However, the raw-data of this capture shown that the RT-PCR positive swabs were collected and analyzed intercalated with RT-PCR negative swabs, suggesting that laboratory cross-contamination is unlikely.

### Temporal variation of seroprevalences

The first attempt to define the temporal dynamic infection of serotine bat colonies was undertaken through a one-year study [86]. In this latter study, seroprevalence declined from 74% to below 10% within a few months (from spring to fall). In contrast, Sera-Cobo at al. (2013) found a significantly higher antibody prevalence in summer when maternity colonies are present in most localities. This pattern was magnified by the presence of multi-species colonies compared to mono-specific colonies, with social contacts between bats. Colony formation, conferring thermodynamic and social advantages to reproductive females during pregnancy and lactation, could indeed increase the rate of rabies exposure due to hypothetically higher probabilities of inter-individual and inter-species interactions. In our study, site A data revealed higher prevalence in summer than in spring, supporting the conclusion that numerous inter-individual interactions of the colony during the post-partum season (care for the juveniles) could increase the probability of exposure.

On site A, corrected seroprevalence decreased over time with significantly higher seropositive frequencies in 2010 (34% of seropositive bats) than in 2011–2013, while on site B, a peak of infection was observed in 2013 (70% of seropositive bats), midway through the 5-year-study. Our data on serotine colonies thus appear to confirm the cyclic temporal hypothesis of bat infections already proposed for *Myotis Myotis*, with an estimated 2–3 year cycle for site A at the time of the study [73]. The model suggests that after the initial introduction of EBLV-1 into the susceptible bat colony, the seroprevalence of the colony increases then, depending on the period, tends to oscillate, its amplitude decreasing year after year.

Ecological studies performed in the bordering of Luxembourg and Germany, a hundred kilometers far from the study area, have shown that females were forming maternity colonies at the middle of April and had a philopatric behavior, meaning that each year the breeding colony invests the same maternity site [72]: This eight years study also shown that median period of birth was happening in the middle of June. The young bats usually make their first flights at around three weeks old, and at six weeks they can forage for themselves. Breeding colonies usually disperse by early September, although a few bats may use the colony site as a roost until early October [72]. Reproduction seems to take place in the autumn, but very little is known about the mating behavior. Hibernation of serotine bats occurs between October and end of March. However, very few information is indeed known about this period. Based on RABV rabies model transmission in the United States, the potential impact of hibernation on the virus’s capacity to remain in animals populations has been raised [74]. The hypothesis is that hibernation could allow infected individuals and their pathogens to survive, infected virus particles being potentially hosted and preserved in brown fat [87]. The relationship between the incubation period, hibernation season and annual birth pulse could indeed generate complex dynamics that should attract more attention in bat rabies studies. With a long incubation period, infected bats could survive long enough to enter hibernation and be responsible for infectious contacts in the main transmission season that follows, maintaining a reservoir until the birth pulse provides a new supply of immunologically naïve bats. Further model predictions fitted this assumption and showed that adult female bats were infectious earlier in the year, whereas infectious juveniles appeared later in the summer [74].

### Characterization of transmission by age

In a previous study, female serotine bats were shown to be more exposed to EBLV-1 than males, probably due to their gregarious social behavior, males being more solitary [88]. Similar findings were also highlighted in the framework of RABV transmission in Brazilian free-tailed bats [89] big brown bats [77] and in vampire bats [90]. In our study, occurring in breeding colonies, only females were assessed and we have shown evidence from site B that seroreversions were significantly more frequent than seroconversions. The seroreversion frequencies of adult females were higher than those of other transition states in juvenile females. Hence, adult female serotine bats appear to be a good indicator of EBLV-1 epizootic dynamics. This raises the question of whether adult female bats are more exposed to the virus due to the mating period in September and whether they could play a major role in virus maintenance, acting as a potential source of virus transmission. Because the reproductive status of adult females could potentially drive inter-individual exposure and transmission differently, it would be valuable to consider the reproductive status as ‘pregnant’, ‘lactating’ and ‘non reproductive’ for further studies. However, such age-related rabies dynamics we detected could also reflect the greater difficulties in characterizing EBLV-1 dynamics in juveniles due to their lower occurrence than adults in the population. The possibility of maternal antibodies transfer in juveniles via the placenta or during lactation also raises questions. Indeed, although this phenomenon has been known and measured for in experimental animals or domestic animals [91–93], the situation regarding bats and EBLV-1 is unknown. Its impact on the antibody level in juveniles, and therefore, on the evaluation of exposure, would need to be clarified. All the EBLV-1 cases detected on sites A and B were detected from end of June to start of August and all determined in dead bats identified morphologically as juveniles (E. Picard-Meyer, under revision), again raising the question of the key role of age in the virus’s transmission. The influx of susceptible young in summer could act as a crucial driver of EBLV-1 dynamics. The role of susceptible young in transmission dynamics has indeed already been raised in previous discussion on zoonotic diseases [94, 95]. Such biological enigmas need to be clarified and additional studies are still needed, especially in the framework of age-related bat EBLV-1characterization.

### Health risk and issues

The means and rate of bat-to-bat transmission in serotine populations still need to be clarified. This is a difficult question to solve in field studies and the legal status of bats in Europe due to the decline of bat populations (all the 36 species are strongly protected by European regulation (Council Directive 92/43/EEC 1992) [96]) has made experimental studies difficult to implement. Only one experimental study of EBLV-1 infection in caged serotine bats was carried out in 2009 through different means of inoculation [97]. It appeared that the environmental contamination of bats is unlikely as none of the intranasally-inoculated bats seroconverted. Infection through bites was indicated as having the greatest potential for inter-bat transmission, as the subcutaneous route of inoculation was found to be relatively efficient [97].

Despite living in close proximity to humans, human contacts with serotine bats are rarely reported. It should be noted that during this study, despite the discovery of two infected bat colonies, no sanitary incidence has been reported, nor in human neither in domestic animals. To date, only 2 EBLV-1 induced human deaths have been reported in Voroshilovgrad, Ukraine (1977) and in Belgorod, Russia (1985) [98]. Experimental infections have also shown evidence of a very limited risk of an EBLV spillover from bat to fox [99]. A few natural EBLV-1 spillover cases have been so far reported in a sheep, a stone marten, 2 cats and a fruit bat [29, 100]. The risk of transmission to other species thus appears very low. However, to avoid any risk of contamination, protective measures such as personal protective equipment, post-exposure rabies prophylaxis or a booster dose in the event of exposure have been established for bat biologists in Europe [96] and France [101, 102].

EBLV-1 antibody carriage in serotine bats was not correlated with mortality probability. In both site A and B, peak-seroprevalences (34 and 70% respectively) were detected one or two years after the first detection of EBLV-1 positive carcasses. While we detected oscillation seroprevalences in time, at annual level, seroprevalences were found higher in summer compare to spring, suggesting that rearing period could increase virus circulation. We pointed out differences of serological statuses between adult female and juveniles and the need for further assessment. A better understanding of this mechanism, whether of ecological, biological and/or immunological origin, is indeed a real challenge and of great interest as elucidating zoonotic virus persistence in bats concomitant to unaffected survival could help to solve human health challenges.

## Supporting information

S1 Table Serological histories observed during the study. (DOCX)
