# Supplementary material for: Longitudinal survey of two serotine bat (Eptesicus serotinus) maternity colonies exposed to EBLV-1 (European Bat Lyssavirus type 1): Assessment of survival and serological status variations using capture-recapture models
Source: PLoS Negl Trop Dis. 2017 Nov 17;11(11):e0006048. doi: 10.1371/journal.pntd.0006048 (PMC5693283; doi:10.1371/journal.pntd.0006048)
Supplement: S1 Table — (DOCX) [file pntd.0006048.s001.docx]

**Supporting Information**

Serological histories observed during the study:

| n individuals site A | n individuals site B | Serological | histories |  |  |  |  |  |  |  |
| --- | --- | --- | --- | --- | --- | --- | --- | --- | --- | --- |
| 31 | 27 | POS |  |  |  |  |  |  |  |  |
| 79 | 57 | NEG |  |  |  |  |  |  |  |  |
| 15 | 21 | INC |  |  |  |  |  |  |  |  |
| 9 | 9 | POS | POS |  |  |  |  |  |  |  |
| 10 | 10 | POS | NEG |  |  |  |  |  |  |  |
| 2 | 3 | POS | INC |  |  |  |  |  |  |  |
| 3 | 4 | NEG | POS |  |  |  |  |  |  |  |
| 21 | 19 | NEG | NEG |  |  |  |  |  |  |  |
| 5 | 9 | NEG | INC |  |  |  |  |  |  |  |
| 2 | 4 | INC | POS |  |  |  |  |  |  |  |
| 7 | 3 | INC | NEG |  |  |  |  |  |  |  |
| 3 |  | INC | INC |  |  |  |  |  |  |  |
| 2 | 2 | POS | POS | POS |  |  |  |  |  |  |
|  | 1 | POS | POS | NEG |  |  |  |  |  |  |
| 1 | 1 | POS | POS | INC |  |  |  |  |  |  |
| 1 | 1 | POS | NEG | POS |  |  |  |  |  |  |
|  | 2 | POS | NEG | NEG |  |  |  |  |  |  |
|  | 2 | POS | INC | NEG |  |  |  |  |  |  |
| 1 | 3 | NEG | POS | NEG |  |  |  |  |  |  |
| 4 | 6 | NEG | NEG | NEG |  |  |  |  |  |  |
| 1 |  | NEG | NEG | POS |  |  |  |  |  |  |
| 1 | 1 | NEG | INC | INC |  |  |  |  |  |  |
| 1 |  | NEG | INC | POS |  |  |  |  |  |  |
| 1 |  | NEG | INC | NEG |  |  |  |  |  |  |
| 1 | 2 | INC | POS | POS |  |  |  |  |  |  |
| 2 | 1 | INC | NEG | NEG |  |  |  |  |  |  |
| 1 |  | INC | INC | NEG |  |  |  |  |  |  |
| 1 | 1 | POS | POS | POS | POS |  |  |  |  |  |
|  | 3 | POS | POS | NEG | NEG |  |  |  |  |  |
| 1 |  | POS | POS | NEG | POS |  |  |  |  |  |
|  | 1 | POS | NEG | POS | NEG |  |  |  |  |  |
|  | 1 | POS | NEG | NEG | NEG |  |  |  |  |  |
|  | 1 | NEG | POS | INC | POS |  |  |  |  |  |
|  | 2 | NEG | NEG | POS | NEG |  |  |  |  |  |
|  | 1 | NEG | NEG | POS | INC |  |  |  |  |  |
| 1 | 2 | NEG | NEG | NEG | NEG |  |  |  |  |  |
|  | 1 | NEG | NEG | INC | POS |  |  |  |  |  |
|  | 1 | NEG | INC | INC | NEG |  |  |  |  |  |
|  | 1 | INC | INC | NEG | POS |  |  |  |  |  |
|  | 1 | INC | INC | NEG | NEG |  |  |  |  |  |
|  | 2 | POS | POS | INC | POS | INC |  |  |  |  |
|  | 1 | POS | NEG | NEG | NEG | INC |  |  |  |  |
| 1 |  | POS | POS | POS | POS | POS |  |  |  |  |
|  | 2 | POS | INC | POS | POS | POS |  |  |  |  |
|  | 1 | NEG | POS | POS | POS | POS |  |  |  |  |
|  | 1 | NEG | POS | NEG | POS | INC |  |  |  |  |
|  | 1 | NEG | POS | NEG | NEG | NEG |  |  |  |  |
| 1 |  | NEG | NEG | NEG | NEG | NEG |  |  |  |  |
|  | 2 | NEG | NEG | INC | NEG | NEG |  |  |  |  |
|  | 1 | NEG | INC | NEG | NEG | NEG |  |  |  |  |
|  | 1 | INC | NEG | NEG | NEG | NEG |  |  |  |  |
|  | 1 | INC | INC | NEG | NEG | NEG |  |  |  |  |
|  | 1 | POS | POS | POS | INC | POS | POS |  |  |  |
|  | 1 | POS | INC | POS | POS | POS | POS |  |  |  |
|  | 1 | NEG | NEG | NEG | NEG | NEG | NEG |  |  |  |
|  | 1 | NEG | NEG | INC | NEG | NEG | NEG |  |  |  |
|  | 1 | NEG | INC | NEG | NEG | NEG | INC |  |  |  |
|  | 1 | INC | INC | POS | POS | POS | POS |  |  |  |
|  | 1 | POS | POS | POS | NEG | INC | NEG | NEG | NEG | NEG |
